# Conservatism and Radicalism in Matters of Dental Education

**Published:** 1888-08

**Authors:** 


### CONSERVATISM AND RADICALISM IN MATTERS OF DENTAL EDUCATION.

The foregoing may be a radical stand to take, nevertheless we think that when individual interest is left out of account, all will admit it to be correct. Those who stand on the side of advance and morality always have the best position. "Easy does it, guv'nur," is the motto of the obstructionist. The foes of progress are always advising us to "go easy," and ofttimes they are so anxious lest we may mar something that they really over-exert themselves trying to restrain the radicals, as they call those who advocate advanced ideas.

Conservatism is often good in business and in politics, but we fail to see its applicability in matters of education. The question, Is it expedient at present to extend the time required to obtain a dental degree? is only another way of asking, Can we afford to take such a stand, knowing that unless it is generally adopted it will cut down the number of our students? The word "expedient" is therefore to be applied only to the interests of the few, and whenever such an argument is advanced, it ought to be understood in the right way.

Conservatism in the question of higher education means forethought for individual interests, and not a care for the best good of the public. Selfishness and individual interests are the main springs of human existence.

Our college faculties are conservative, because they are private institutions, and as such, the only way to control them is through

State laws. True, we have high-minded men in the profession who are willing to sink individual interests for the general good. But it is equally true that most of our colleges are conducted on purely business principles, and that the pocketbook is consulted rather than the good of the profession. And again, just in proportion to the interests at stake—that is, the larger the number of students a college has—so will that college be slowest in adopting any measure which looks towards reducing the number of students. Here is an example of what we have just said : A resolution was brought up by a junior member in the faculty of one of our most prosperous colleges, looking towards extending the course of instruction to two years, of seven months each.

The motion was lost however, because of the opposition of one of the oldest members of the faculty, who argued that they could not afford to take the lead in the matter. The question was lost, not on the ground that the increase is not needed, but because the faculty feared that their revenue might be reduced, and therefore it was not "expedient." It is time that the profession at large demand that our colleges be conducted in the interest of the profession, and not in the interest of the few. The only way to strike at the root of the evil is to see that no person connected with educational institutions is appointed upon our Boards of Dental Examiners. But, it is argued, the class of students presenting themselves is not capable of meeting the requirements of a more rigid examination. "You cannot make silk purses out of sow's ears," is a favorite saying of one of our most prominent educators. What is wanted is to weed out the "sow's ears." The profession can do much in the matter of selecting the class of students that shall present for matriculation. With regard to the standard of medical matriculants, Dr. J. E. Garretson says, A full one-half of the young men who come to Philadelphia to study medicine should be turned "about face," and sent to a village school."* Another writer † says, "The place to intercept incompetents is at the entrance of medical schools, not at the end. The profession is not thoroughly awakened to the necessity of arresting the course of the schools that are annually sending forth thousands of improper persons to practice on the community. The diploma of schools should be beyond sus-

* Exchange.

† New England Med. Monthly.

picion. The signatures should do more than convey the intelligence that the holder of a certain certificate has paid his money for a course of lectures and thirty dollars for the engrossed parchment attesting same. It does little more just now."

We fully concur with Prof. Garretson upon this point. But if such is the condition of those who apply for admission into our medical schools, what must be the proportion of dental applicants that should be returned to their preliminary studies? The time to begin the reformation is at the selection of matriculants, and the time for its completion is when the final examinations are made.

Unless the colleges adopt some uniform rule regarding time and attainments required for graduation, the matter will be taken up by the different States. Thirty-four States have at present statutory enactments looking towards the regulation of dental practice. These enactments are becoming more and more rigid as the people are better informed regarding the need of such laws.

